# Translation and equivalence assessment for a Japanese version of the modified Parental Nurturance Scale: a comparative study

**DOI:** 10.1186/1751-0759-1-4

**Published:** 2007-01-22

**Authors:** Chizu Mimura, Peter Griffiths

**Affiliations:** 1 Florence Nightingale School of Nursing and Midwifery, King's College London, Waterloo Road, London, SE1 8WA, UK

## Abstract

**Background:**

This paper reports on the modification of the Parental Nurturance Scale (PNS), translation of the modified version (PNSM) from English to Japanese, and equivalence assessment between the PNSM and the translated version (PNSM-J). The PNS was modified so as to enable its use in nurturance studies where the prime source of nurturance might vary between respondents.

**Method:**

It was translated into Japanese through the forward-backward translation procedure. With attempting to enhance representativeness of language in the target populations, translators used were married couples that consisted of a native English speaker and a native Japanese speaker. Multiple translations were produced and used to make a single Japanese version. A panel of reviewers identified problems in conceptual and semantic equivalence between the original and the translated versions. The Japanese version was altered accordingly with reference to alternate Japanese forms from the original English to Japanese translations. The altered translation was again re-translated into English and problematic differences were checked. This forward-backward process was repeated until satisfactory agreement was attained. The PNSM was administered to 222 native English speakers and the PNSM-J to 1320 native Japanese speakers.

**Results:**

Factor analysis and target rotation revealed a nearly identical factor structure and factor loadings of the items of the PNSM and PNSM-J between the different cultural groups. High Cronbach's alpha coefficient supported the reliability of the test scores on both versions.

**Conclusion:**

The equivalence between the two scales was supported. It is suggested that the PNSM and PNSM-J are suitable tools for comparative cross-cultural studies.

## Background

It is widely recognised that nurturing from significant others, especially parents, play an important role in the development of adult personality and self-concept [1-4]. A number of theories which advocate this conception have been developed, for example, Transactional Analysis [5], Self-esteem Formation Theory [6], Adult Children Theory [7], the Theory of Human Motivation [8] and the True Self and False Self Theory [9]. Specifically, these theories suggest that positive affirmation and caring from parents or a parental substitute, 'nurturance', are crucial in the formation of positive self-esteem. However, there have been relatively few empirical works on the issue of parenting, in which the respondents' perception of the nurturance provided by their significant others was measured.

The PNS was developed by Buri, Kirchner and Walsh to examine the point of view of an individual regarding the parental nurturance he/she received [10]. The scale is answered on a five-point Likert format. Each item is rated from 1 = strongly agree to 5 = strongly disagree but the items stating positive attitudes require reverse scoring. In constructing the original PNS, content validation analyses were conducted by using numerous parental nurturance sources [11]. Good reliability has been reported with Cronbach's alpha of 0.95 for mothers' nurturance and 0.93 for fathers' nurturance, and two-week test-retest reliability of 0.92 for mothers' nurturance and 0.94 for fathers' nurturance [10].

The PNS has been mainly used for studying the relationship between parental nurturance and self-conception [12] and self-esteem development [10,11,13]. These studies show that parental nurturing far outweighs other familial factors such as parental self-esteem in influencing self-esteem development [10] and that although the strength of relationship declines over time, it remains a strong predictor into early adulthood [13].

The PNS has two versions, paternal and maternal nurturance scales. These are phrased as either "my father believed in me" or "my mother believed in me." Consequently, use of the PNS is necessarily confined to studies exploring nurturance where parents are pre-defined as the most important sources of rearing and where, unless multiple questionnaires are to be used, a single parental role is the focus. Modification was, therefore, attempted so that the scale can be utilised in studying treatment received from any significant other.

Although there has been a growing interest in the issue of parental nurturance within psychological research in Japan, there do not seem to be validated translated Japanese versions of the PNS or other instrument measuring the specific phenomenon of nurturance. An alternative instrument, the Parental Bonding Instrument (PBI) [14] is probably the most widely used measure of the parent-child relationship, and a Japanese version does exist [15]. However, the PBI does not focus exclusively on the parent's behaviour and appears to measure a number of different construct such as 'over protection' in addition to a 'caring' construct that may be equivalent to 'nurturance.' Further, the majority of studies using it have focused upon the development of depressive conditions, not self-esteem. Its factor structure remains uncertain, with continuing dispute over the number of factors, relationships between factors [16,17] and question as to whether or not the instrument measures actual as opposed to perceived parental behaviour [18].

Thus, for the purpose of future cross-cultural comparative studies, it would be of value to have properly translated PNS and to examine reliability, validity and equivalence. This paper reports on the modification of the Parental Nurturance Scale (PNS) [11], translation of the modified version (PNSM) from English to Japanese, and equivalence assessment between the PNSM and the translated version (PNSM-J). Permission to modify the PNS and translate it into Japanese was gained from the creator, Professor J. R. Buri. The study was conducted under the aegis of a wider study, which had been ethically scrutinised and approved by the authors' institutional ethical committee.

## Method

### Modification

In order for the scale to be able to be used for other significant persons, the item statement of "my father" or "my mother" was altered to "the person", for example, "the person believed in me." Prior to answering the questions, respondents need to identify a person who they are going to rate as the most significant source of nurturance. With this alteration, the scale became usable for examining nurturance of any persons.

The PNS originally consisted of 24 items, however 6 items appeared to convey two different but close meanings. Such items were divided into two items in the present study in order to avoid possible confusion of respondents. For example, "the person consoled me and helped me when I was unhappy or in trouble" became "the person consoled me when I was unhappy" and "the person helped me when I was in trouble." This method yielded 30 items in total. Consequently, a possible scoring was from 30 to 150 with higher scores indicating a higher level of acceptance. Since the number of items was changed, the item ordering was determined by using a table of random digits.

### Translation

This study was undertaken as part of a larger study which developed Japanese versions of a number of scales including PNSM at the same time with the same translation method. In relation to another scale, a preliminary testing study was published elsewhere [19], which described the translation approach and discussed the issues concerning cross-cultural translation in detail. The translation procedures were informed by the European Research Group on Health Outcome recommendations [20] and the International Test Commission Guidelines [21]. The repeated forward-backward translation procedure was adopted as the most suitable strategy that was pragmatically possible.

In Phase 1, four married couples of British and Japanese origin were separately asked to translate the original scale into Japanese with each couple among themselves discussing the conceptual, semantic and content equivalence between the original and their translation. The four couples were selected in accordance with the following criteria:

(1) one member of the couple was a native English speaker and the other a native Japanese speaker;

(2) both members were reared and educated either in English in an English-speaking country or in Japanese in Japan until at least 18 years of age;

(3) they have spent more than five years together since they married.

These criteria were used to identify translators who were familiar with both their own language and cultural background and that of the alternative language. The use of married couples was based on the opportunity such couples presented for exchanging a native speaker's insight into expressions in different languages among an intimate couple without the bias introduced by restricting translators to those with a formal academic training in language translation, whose usage may not be typical of the population as a whole. None of the individuals involved were professional translators. Thus it was hoped that an equivalent translation would be produced that was potentially more representative of the wider cultures than would be gained from a bilingual person or highly trained translators. All four couples happened to be of a British male and a Japanese female. They were fully informed of the objectives of their role in the whole procedure and were asked to discuss conceptual, semantic and content equivalence and to emphasise meaning rather than word-to-word translation. One of the authors (CM whose first language is Japanese) unified the four Japanese translations created by this process into a single translated version. Selection among alternative Japanese translations was based upon the perceived "naturalness" of linguistic expression in the Japanese language version.

In Phase 2, an additional couple was identified using the same criteria. They were asked to back-translate the Japanese version produced in Phase 1 without sight of the original version. In Phase 3, five university lecturers at the authors' college (native English speakers) compared the original scale and the back-translation brought about by Phase 2, and checked for semantic discrepancies. In Phase 4, the author altered the Japanese expression of the parts found to be problematic in Phase 3 with reference to any alternatives rejected in Phase 1. The couple used in Phase 2 re-translated them into English. One of the panel used in Phase 3 checked discrepancies between the original scale and the re-translation. Detailed discussion of cultural difference and nuance aimed to ensure semantic equivalence and to overcome conceptual differences by identifying parallel concepts. This process was repeated until problems were resolved.

### Equivalence assessment

#### Respondents

Data were collected in the UK using the modified English language PNSM and in Japan using the translated version which we refer to as the PNSM-J. Subjects were recruited from full-time BSc nursing and pharmacy students of all years (1 to 4) at single university institutions in central London and Tokyo. Non-native English/Japanese speakers were excluded as appropriate to the version of the scale being tested. Data were obtained from 131 nursing and 91 pharmacy students in the UK (n = 222) of whom 194 were female (87.4%). Ages ranged from 18 to 45 and the mean age was 22.05 (*SD *= 4.51). The Japanese sample comprised 344 nursing and 976 pharmacy students (n = 1320) of whom 1018 were female (77.1%). Ages ranged from 18 to 44 and the mean was 20.58 (*SD *= 2.80). There were small but statistically significant differences between profession within both countries with a lower proportion of females in pharmacy group (Table 1). However, females were the vast majority in all professional groups in both countries with the smallest proportion being among Japanese pharmacists (71%). Nurses were significantly older than pharmacists in Japan but the mean difference, less than one year, was small. The proportion of females was significantly higher in the UK sample as was the mean age. Again, the differences were small in absolute terms (Table 1).

**Table 1:** Profile variables

			*Nursing*	*Pharmacy*	*Difference*
UK	Age	n	131	91	
		Mean (SD)	22.01 (4.79)	22.10 (4.08)	t = .148 df = 220 p = 0882
	Female	n (%)	120 (92%)	74 (81%)	χ^2 ^= 5.15 df = 1 p = .023

Japan	Age	n	344	976	
		Mean (SD)	21.28 (3.21)	20.34 (2.60)	t = 4.88 df = 1305 p < .001
	Female	n (%)	328 (96%)	690 (71%)	χ^2 ^= 85.3 df = 1 p < .001

			*UK*	*Japan*	*Difference*

All	Age	n	122	1320	
		Mean (SD)	22.05 (4.51)	20.58 (2.80)	t = 4.682 df = 1528 p < .001
	Female	n (%)	194 (87%)	1018 (77%)	χ^2 ^= 11.22 df = 1 p = .001

#### Data collection

The questionnaire was administered to the students in a class setting. After permission for access to the students was obtained from the head of department and the course leader, the investigator visited the class in a room before or after a lecture. The questionnaires were distributed only to students who agreed to participate in the study. For the pharmacy students in the UK, it was not possible for all students to complete the questionnaires immediately owing to their tight academic time schedule. Therefore, a designated box was allocated in their school, and they could choose to complete the questionnaire immediately or to return it in the box later. For all other groups questionnaires were gathered in the envelopes provided immediately after they finished completing the questionnaire in the room.

#### Analysis

Factor structure was assessed by using exploratory factor analysis of principle component method. For both the PNSM and PNSM-J, only one factor was extracted, and therefore it could not be rotated. As the prime aim here was to assess equivalence, confirmatory factor analysis was not performed for the uni-factorial structure. Instead, factors were compared with target rotation as suggested by Van de Vijver [22]. The factor of the PNSM was rotated to the loadings of the PNSM-J. This was to assess the construct equivalence between the PNSM and PNSM-J, that is, the extent to which the loadings on the factor in the different culture groups were identical. Cronbach's alpha coefficient was calculated to examine internal consistency reliability of the data for the PNSM and PNSM-J.

## Results

### Factor structure

The adequacy for the data to be factor analysed was examined by diagnostic tests in the first place. Bartlett's test of sphericity showed a significant result (p < 0.001) for both the PNSM and PNSM-J. The Kaiser-Meyer-Olkin test (KMO) was 0.96 for both the PNSM and PNSM -J, and the individual measure of sampling adequacy (MSA) ranged from 0.93 to 0.97 and from 0.90 to 0.98 respectively. Pett, Lackey, and Sullivan have argued that the KMO and MSA of greater than 0.6 represent acceptable results [23]. Thus the data were considered to be adequate for proceeding factor analysis.

The distribution of the initial eigenvalues (Table 2) was scrutinised next. There were four factors with an eigenvalue greater 1 for both the PNSM and PNSM-J. However, the eigenvalue of the first largest factor was 16.06 and of the second and smaller factors were 1.44, 1.15 and 1.08 in order regarding the PNSM. Similarly, these eigenvalues were 12.18, 1.76, 1.53 and 1.27 respectively for the PNSM-J. The differences in eigenvalues between the first and second largest factors were remarkably large compared to the rest. Also, the largest factor accounted for 53.5% of the variance in the PNSM and 40.59% in the PNSM-J, but lesser factors accounted only for a few percents each. These findings suggested that there were actually only one significant construct.

**Table 2:** Initial eigenvalues explained by factors

	*PNSM *^*a*) ^*(n = 222)*			*PNSM-J *^*b*) ^*(n = 1320)*		
		
*Component*	*Total*	*% of Variance*	*Cumulative %*	*Total*	*% of Variance*	*Cumulative %*
		
1	16.06	53.53	53.53	12.18	40.59	40.59
2	1.44	4.81	58.35	1.76	5.85	46.45
3	1.15	3.82	62.17	1.53	5.09	51.54
4	1.08	3.60	65.77	1.27	4.22	55.76
5	0.93	3.11	68.88	0.99	3.31	59.07
6	0.82	2.74	71.62	0.84	2.82	61.89
7	0.72	2.40	74.02	0.79	2.62	64.51
8	0.68	2.26	76.28	0.76	2.54	67.04
9	0.61	2.04	78.32	0.71	2.37	69.41
10	0.56	1.87	80.19	0.65	2.16	71.57
11	0.53	1.77	81.96	0.61	2.04	73.61
12	0.49	1.62	83.57	0.59	1.95	75.56
13	0.46	1.52	85.10	0.58	1.93	77.49
14	0.44	1.45	86.55	0.54	1.81	79.30
15	0.41	1.36	87.91	0.53	1.76	81.06
16	0.38	1.27	89.18	0.50	1.67	82.73
17	0.34	1.15	90.33	0.49	1.63	84.36
18	0.33	1.11	91.44	0.46	1.54	85.89
19	0.32	1.08	92.52	0.45	1.53	87.42
20	0.30	1.00	93.52	0.43	1.42	88.84
21	0.28	0.95	94.47	0.41	1.38	90.22
22	0.26	0.85	95.32	0.39	1.28	91.50
23	0.23	0.77	96.10	0.38	1.26	92.77
24	0.21	0.71	96.81	0.35	1.15	93.91
25	0.20	0.66	97.47	0.33	1.09	95.00
26	0.18	0.61	98.08	0.32	1.07	96.07
27	0.16	0.54	98.63	0.31	1.06	97.13
28	0.15	0.52	99.15	0.30	0.98	98.11
29	0.14	0.47	99.62	0.29	0.97	99.08
30	0.11	0.38	100.00	0.28	0.92	100.00

NOTE: Extraction method – principal component analysis

a): The modified Parental Nurturance Scale, administered to native English speakers

b): Translated Japanese version of the modified Parental Nurturance Scale, administered to native Japanese speakers

Item loadings on the largest factor were generally very high (Table 3). All the items in the PNSM and two thirds of items in the PNSM-J exceeded 0.6. For the PNSM, all the items correlated most highly with the first largest factor. As for the PNSJ, only two items, Items 6 and 13, were most highly correlated with the second factor (0.55 and 0.58 respectively), but these two items also indicated high correlation with the first factor. All other items were most highly correlated with the first factor.

**Table 3:** Factor loading, factorial agreement and reliability coefficient

Item			*PNSM *^*a*) ^*(n = 222)*	*PNSM-J *^*b*) ^*(n = 1320)*	*Difference in Loading*
	*Statement*	*Attitude*	*Factor I*	*Factor I*	
			
1	Nothing I did ever seemed to please the person.	-	0.70	0.47	0.23
2	The person enjoyed spending time with me.	+	0.70	0.62	0.08
3	The person did not really know what kind of person I was.	-	0.70	0.55	0.15
4	The person was a caring individual.	+	0.73	0.45	0.28
5	The person was removed when I was with him/her.	-	0.64	0.61	0.03
6	The person took an active interest in my affairs.	+	0.73	0.50	0.23
7	The person consoled me when I was unhappy.	+	0.80	0.67	0.13
8	The person was very understanding.	+	0.83	0.68	0.15
9	The person was a warm individual.	+	0.81	0.72	0.09
10	The person did not feel that I was interesting.	-	0.71	0.57	0.14
11	The person believed in me.	+	0.79	0.55	0.24
12	The person seldom showed me any affection.	-	0.75	0.72	0.03
13	The person was very interested in those things that concerned me.	+	0.73	0.49	0.24
14	The person was very sympathetic.	+	0.76	0.67	0.09
15	I was tense and/or uneasy when the person and I were together.	-	0.68	0.65	0.04
16	The person did not feel that I was important.	-	0.68	0.69	-0.01
17	The person often acted as if he/she did not care about me.	-	0.72	0.57	0.15
18	The person expressed his/her warmth and/or affection for me.	+	0.78	0.70	0.08
19	The person was easy for me to talk to.	+	0.78	0.64	0.14
20	I was an important person in the person's eyes.	+	0.69	0.67	0.02
21	I did not feel that the person enjoyed being with me.	-	0.76	0.67	0.09
22	The person did not really care much what happened to me.	-	0.61	0.57	0.04
23	I feel that the person found fault with me more often than I deserved.	-	0.69	0.56	0.13
24	The person was often critical of me.	-	0.58	0.66	-0.08
25	The person seldom said nice things about me.	-	0.77	0.68	0.09
26	The person was generally cold when I was with him/her.	-	0.82	0.74	0.08
27	I felt very close to the person.	+	0.75	0.73	0.02
28	I received a lot of affirmation from the person.	+	0.76	0.74	0.02
29	The person helped me when I was in trouble.	+	0.67	0.71	-0.04
30	The person did not understand me.	-	0.76	0.71	0.05
% of Variance			53.5	40.6	N/A

	Target Rotation	Identity coefficient	0.98		N/A
		Proportionality coefficient	0.99		N/A

Cronbach's alpha coefficient			0.97	0.95	N/A

NOTE: Extraction method – principal component analysis

a): The modified Parental Nurturance Scale, administered to native English speakers

b): Translated Japanese version of the modified Parental Nurturance Scale, administered to native Japanese speakers

The target rotation revealed that the loadings of each item were very similar between the PNSM and PNSM-J. The identity coefficient was 0.98, and the proportionality coefficient was 0.99. The differences in item loading were largely very small between the two scales, with almost all items indicating a difference less than 0.2. The results of the factor analysis and target rotation are reported in Table 3.

### Internal consistency reliability

Cronbach's alpha coefficient of the data from the PNSM was 0.97 (95% CI = 0.96 to 0.97) and PNSM-J was 0.95 (95% CI = 0.94 to 0.95). McColl, Christiansen, and König-Zahn have suggested that it is generally considered to be acceptable if the figure is in excess of 0.7 for a scale [24]. Therefore, these results were satisfactory high. These results are also presented in Table 3.

### Differences by significant other

The person most frequently identified as significant other was mother in both UK and Japanese samples (UK n = 185, 83.3%; Japan n = 1085, 82.2%). In the UK sample, father was the second most frequent person identified (n = 20, 9.0%). In the Japanese sample, the next most frequent significant other was brother/sister (n = 63, 4.8%) followed by grandmother (n = 53, 4.0%). Father was the fourth (n = 41) being identified by only 3.1% of the subjects.

As mothers and fathers are the only category that appears frequently enough in both samples for comparison and because previous work has shown differences between ratings of fathers and mothers [12,25-27], the mean for these groups were compared. In the UK sample, the mean nurturance score for mother was 126.6 (SD = 21.6) and for father 117.8 (SD = 21.0). The mean difference of 8.8 between the two was not statistically significant (t = 1.739, df = 203, p = 0.084, CI = -1.184 to 18.849). In the Japanese sample, the mean nurturance score for mother was 123.2 (SD = 16.1) and 114.0 (SD = 21.2) for father. This difference (9.2) was statistically significant (t = 2.755, df = 41.771, p = 0.009, CI = 2.463 to 15.969). The results are presented in Table 4.

**Table 4:** Mean difference by carer

	*PNSM *^*a*)^		*PNSM-J *^*b*)^	
		
*DESCRIPTIVES*	*n = 222*		*n = 1320*	
		
*Carer*	*Frequency (%)*	*Mean (SD)*	*Frequency (%)*	*Mean (SD)*
Mother	185 (83.3)	126.6 (21.6)	1085 (82.2)	123.2 (16.1)
Father	20 (9.0)	117.8 (21.0)	41 (3.1)	114.0 (21.2)
Parents	4 (1.8)	123.0 (14.6)	12 (0.9)	122.1 (17.4)
Grandmother	2 (0.9)	147.0 (4.2)	53 (4.0)	125.2 (17.2)
School teacher	2 (0.9)	102.5 (34.6)	8 (0.6)	118.8 (11.0)
Nanny	2 (0.9)	111.5 (31.8)	0	
Brother/Sister	1 (0.5)	146.0	63 (4.8)	112.8 (16.2)
Aunt	1 (0.5)	150.0	1 (0.1)	120.0
Foster parent	1 (0.5)	68.0	1 (0.1)	103.0
Friend	1 (0.5)	106.0	34 (2.6)	121.2 (13.5)
Grand father	0		18 (1.4)	122.9 (18.8)
Neighbour	0		2 (0.2)	139.0 (9.9)
Myself	0		1 (0.1)	109.0
Pet	0		1 (0.1)	92.0
Not identified	3 (1.4)		0	

*t TEST (Mother vs Father)*	*n = 205*		*n = 1126*	
		
t	1.739		2.755	
df	203		41.771	
Significance	p = 0.084		p = 0.009	
CI of difference	-1.184 to 18.849		2.463 to 15.969	

NOTE: a): The modified Parental Nurturance Scale, administered to native English speakers

b): Translated Japanese version of the modified Parental Nurturance Scale, administered to native Japanese speakers

## Discussion

In spite of an intensive literature review, no published work was identified that had analysed the factorial nature of the original PNS. More importantly, the PNS was modified in the present study and the number of items changed from 24 to 30. Therefore, there was no model against which to compare the factor structure of the scales used in the present study. The outstandingly large eigenvalues of and predominant percentage of variance explained by the first largest factor for the PNSM and PNSM-J suggested that the both scales were uni-dimensional in factorial nature for the use on the sample of the present study.

Factorial congruence is supported where identity and proportionality coefficients are 0.9 or greater, which has been argued by Van de Vijver [22]. These coefficients obtained in this study were excess of the figure. Additionally, high Cronbach's alpha for each factor supported that scores on both the scales were internally consistent. It can, therefore, be deducted that the PNSM and PNS-J were equivalent for the two groups of the different cultures.

In examining levels of parental nurturance with the PNSM and PNSM-J, mean scores obtained cannot simply be compared to findings in previous research because the number of items has been altered. The original scale consists of 24 items and possible scores are between 24 and 120 while the PNSM and PNSM-J included 30 items with a score range from 30 to 150. Nevertheless, the wording or meaning of the items has not been changed at all. Thus, an approximate equivalent score can be obtained by simply multiplied the scores on the PNSM or PNSM-J by 0.8 (24/30), or an index (%) by using the equation [(mean score - minimum possible score) ÷ (maximum possible score - minimum possible score)]. These would enable some comparison between scores on the original PNS reported in previous studies and on the PNSM and PNSM-J used in future research.

It has been consistently found in previous research that maternal nurturance is higher than paternal nurturance [12,25-27]. In this study, this difference was also suggested although it was not statistically significant in the UK sample. Previous work measured maternal and paternal nurturance in the same person and so direct comparison with this finding is not possible. However, these results along with previous findings emphasise that attention should be paid to who is identified as significant other when levels of nurturance are compared by using PNSM or PNSM-J.

There is a diversity of issues in translating a health-related measurement into another language as equivalent as possible. Most important aspects are conceptual and semantic problems such as differences in conceptualisation and behaviours associated with the construct of a scale, poor comprehensibility and inappropriateness of item content, and loss of natural flow from the use of literal translations or stilted language [20,21]. In order to address these issues, this study conscientiously complied with existing guidelines as described in the translation section. On the other hand, couples of a native English speaker and a native Japanese speaker were used in producing multiple forward and backward translations, which can be said to be an innovative method. This contrivance probably further contributed to the equivalence between the original PNSM and target language version of PNSM-J. However such couples are likely to differ from the population in general. The translation might, therefore, be biased although professional translators and those who generate the original items on such scales are equally unlikely to represent the general population.

The gender imbalance in translation team (ie. Male native English speakers and female native Japanese speakers) and a single person, who is also female, unified the Japanese translation. This might have led to a translation that does not fully present the concept of nurturance from a male perspective. The gender of translators is rarely considered or reported, and in this study could not be manipulated within the resources available. It is an area for future study, but for the moment the possible impact is a matter for speculation. However, the original version of the tool was prepared by a male native English speaker and appears to be equivalent to Japanese version prepared by a female Japanese speaker. Thus, we consider the possibility that it invalidates the scale to be a small one.

Another limitation of this assessment is that the subjects used were convenience sample recruited from a single institution in each country although the sample size was large. The comparison of the samples differed somewhat between countries and groups although the magnitudes of the differences were small. However, all were undergraduate nursing or pharmacy students and the sample was predominantly female. The findings may, therefore, be influenced by factors unique to them such as gender, social status or particular characteristics relating to the subject of study. Further equivalence assessment using a sample that is more representative of the population in general would ideally resolve this limitation.

## Conclusion

The findings of factor analysis and target rotation showed a near identical factor structure and factor loadings of item scores between the modified version of the Parental Nurturance Scale (PNSM) and the translated Japanese version of PNSM (PNSM-J). High and similar internal consistency reliability was also shown. The equivalence between the two scales was supported. It is concluded that the PNSM and PNSM-J are suitable tools for the study of nurturance that people received from their significant others among native English and Japanese speakers in cross-cultural studies examining the interrelationship between nurturance and personality factors such as self-esteem or aspects of adult psychological well-being that may be determined by childhood experiences. The single factor solution for both the PNSM and the PNSM-J suggest that it may be a more suitable instrument than the PBI to use when a single dimension of nurturance is of interest. However, it is certainly important that research using this scale in new population assesses the validity and reliability for its own sample and future studies exploring the relationship between the PNSM/PNSM-J and the PBI would be of value.

## Competing interests

The author(s) declare that they have no competing interest.

## Authors' contributions

CM conceptualised and designed the study, coordinated the translation process, collected and analysed the data, interpreted the results and drafted the manuscript. PG participated in its design and translation, supervised the analysis and interpretation, and helped to draft and revise the manuscript. All authors read and approved the final manuscript.
